# Phenotypic Heterogeneity in Familial Takayasu Arteritis Across Two Families: A Case-Based Review

**DOI:** 10.31138/mjr.120825.atf

**Published:** 2026-04-21

**Authors:** Anisio U.L. Santana, Samuel K. Shinjo

**Affiliations:** Rheumatology Division, Faculdade de Medicina FMUSP, Universidade de São Paulo, São Paulo, SP, Brazil

**Keywords:** case reports, phenotype, systemic vasculitis, Takayasu arteritis

## Abstract

**Background:**

Takayasu arteritis (TAK) is a rare large-vessel vasculitis that predominantly affects young women. Familial cases are uncommon but suggest a possible genetic predisposition.

**Case Report:**

We describe five familial TAK cases from two families. In the first, two sisters had distinct disease patterns: one with severe supra-aortic involvement (Hata IIa), requiring intensive immunosuppression and vascular interventions; the other with isolated abdominal aortic disease (Hata IV), successfully managed with standard therapy. In the second family, three siblings presented with varying severity. One sister had mild disease responsive to therapy, whereas another developed rapidly progressive TAK with multivessel involvement, necessitating complex surgical management and ultimately succumbing to COVID-19 complications. Their brother presented with extensive Hata V involvement and achieved remission following brief medical therapy.

**Conclusion:**

Familial TAK demonstrates marked clinical and radiological heterogeneity, even among first-degree relatives. These findings underscore the importance of clinical vigilance and prompt evaluation for relatives who develop suggestive symptoms, to enable timely diagnosis and improve clinical outcomes.

## INTRODUCTION

Takayasu arteritis (TAK) is a rare primary systemic vasculitis that predominantly affects large-calibre arteries, including the aorta and its main branches.^1^ The disease mainly affects young women.^2,3^ The occurrence of familial TAK is exceptionally rare, with only a few dozen cases documented in the literature.^4–28^ Reported cases include reports of monozygotic twins, siblings, parent-child pairs, and even a family with five affected siblings. These scarce reports suggest a potential genetic predisposition. However, the heritability and clinical spectrum within families remain poorly understood. To contribute to this limited body of knowledge, we describe five cases from familial TAK from two distinct families treated at our tertiary centre and provide a review of the literature.

## METHODS

This descriptive, retrospective case series was conducted at a single tertiary university hospital. The report was prepared in accordance with the CABARET recommendations for case series reporting.^29^

### Patient selection and data collection

The cases were identified from the outpatient vasculitis clinic database of our institution between January 2009 and October 2025. We included patients diagnosed with Takayasu Arteritis who had at least one first-degree relative with a confirmed diagnosis of the same disease. Data were retrospectively collected from electronic and physical medical records. The extracted information included demographic data, clinical presentation, laboratory results (*e.g*., erythrocyte sedimentation rate [ESR], C-reactive protein [CRP]), radiological findings from computed tomography (CT) angiography and magnetic resonance angiography (MRA), treatment regimens (including corticosteroids, immunosuppressants, and biologics), surgical interventions, and clinical outcomes. Disease classification was based on the Hata criteria.

### Literature Review

To contextualise our findings, a literature review was performed using the PubMed, Scopus, Web of Science, Directory of Open Access Journals (DOAJ), SciELO, and Embase databases up to October 2025. The search strategy included the keywords: “Takayasu arteritis,” “familial,” “hereditary,” “siblings,” “twins,” “brothers,” and “sisters.” We prioritised full-text articles in English, but relevant studies in other languages were also considered. The reference lists of included articles were manually scanned to identify additional cases. Conference abstracts and proceedings were excluded.

## CASE DESCRIPTION

### First family (two sisters)

**Figure 1.**
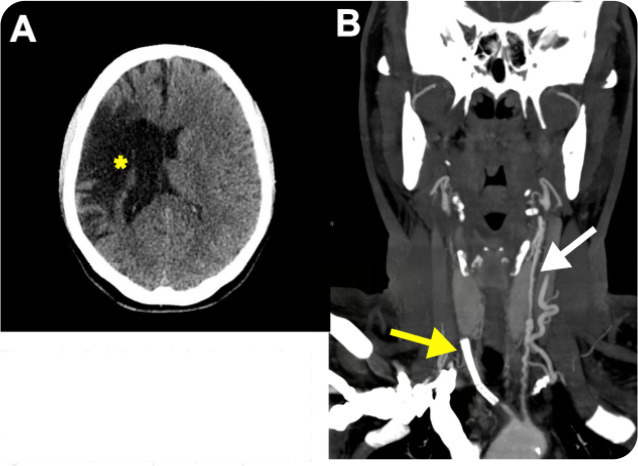
Radiological findings in Case 1 from the first family (the 42-year-old sister). (A) Axial view of a non-contrast brain CT showing a large area of encephalomalacia (yellow asterisk) in the right cerebral hemisphere, a sequela of a chronic ischemic stroke. (B) Coronal view of a CT angiogram of the cervical arteries showing an occluded stent in the right common carotid artery (yellow arrow) and severe stenosis of the left common carotid artery (white arrow).

A 42-year-old woman first developed syncopal episodes at age 21, culminating in a major ischemic stroke at age 27 that resulted in left hemiparesis and aphasia (**Figure 1A**). The subsequent etiological investigation for the stroke revealed absent radial, carotid, axillary, and brachial pulses on physical examination. This prompted a CT angiography in March 2009, which confirmed the diagnosis of TAK by demonstrating concentric mural thickening and stenosis of all supra-aortic branches, with complete occlusion of the right carotid artery and near-occlusion of the left (**Figure 1B, Table 1**). She was diagnosed with TAK, Hata type IIa, and began follow-up at our tertiary centre. Treatment included prednisone 1 mg/kg/day, five monthly pulses of cyclophosphamide (1.0–1.2 g per pulse), and subsequently methotrexate (up to 25 mg/week). Due to refractory symptoms, infliximab was initiated in June 2013 at a dose of 5 mg/kg every six weeks. Treatment was continued for nearly eight years, with dosing interval modifications implemented to maintain disease control, until discontinuation in April 2021 following sustained remission. Due to persistent syncope at the time of diagnosis, stents were placed in the left subclavian and left vertebral arteries, and later in the right brachiocephalic trunk. She also underwent right coronary angioplasty due to presyncope upon cervical extension. The patient has been in remission since 2021, without glucocorticoids, immunosuppressants, or biologics.

**Table 1.** General characteristics of the patients with TAK from the first family.

	**Case 1**	**Case 2**
Age at diagnosis (years)	27	34
Initial manifestations	Syncope	Abdominal pain
Radiological classification (Hata)^30^	IIa	V
Radiological manifestations		
Stenosis		
Left carotid artery	X	-
Right carotid artery	X	-
Left vertebral artery	X	-
Right vertebral artery	-	-
Brachiocephalic trunk	X	-
Left subclavian artery	X	-
Right subclavian artery	X	-
Abdominal aorta	-	X
Occlusions		
Left carotid artery	X	-
Right carotid artery	X	-
Middle cerebral artery	X	-
Thickenings		
Left carotid artery	X	X
Right carotid artery	X	X
Left vertebral artery	-	-
Right vertebral artery	-	-
Brachiocephalic trunk	X	-
Left subclavian artery	X	-
Right subclavian artery	X	-
Abdominal aorta	-	X

Her sister, currently 34 years old, had been experiencing abdominal pain for several months without an apparent cause. An initial abdominal CT scan revealed periaortic inflammation, and a subsequent CT angiography demonstrated concentric thickening of the abdominal aorta. Carotid ultrasonography also showed diffuse intima-media thickening. These findings established the diagnosis of TAK, Hata type V, with involvement of the abdominal aorta, celiac trunk, superior mesenteric artery, and carotid arteries (**Table 1**). It was initiated on prednisone (initially 60 mg/day) and methotrexate, with the dose currently being escalated to 25 mg/week. She continues to experience symptoms upon prednisone tapering, and a biologic agent is planned due to persistent disease activity.

### Second family (two sisters and one brother)

**Figure 2.**
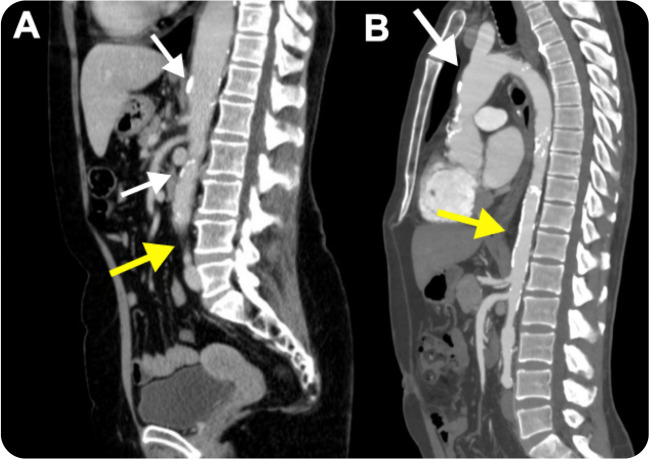
Heterogeneity of aortic involvement in two siblings from the second family. (A) Sagittal view of a CT scan from Case 1 (the 69-year-old sister) showing a different pattern with narrowing of the distal abdominal aorta (yellow arrow) and focal parietal calcifications (white arrows). (B) Sagittal view of a CT angiogram from Case 3 (the 58-year-old brother) showing diffuse parietal thickening and calcification of the aorta (yellow arrow) and ectasia of the ascending aorta (white arrow).

A 69-year-old woman was diagnosed with TAK at age 32, when she presented with lower limb claudication. On examination, she had abdominal and carotid bruits, a systolic murmur, and an absent brachial pulse. Laboratory tests revealed an elevated erythrocyte sedimentation rate. Her diagnosis was established through imaging studies, including aortic CT scan and Doppler ultrasonography, which revealed focal mural calcifications and narrowing of the distal abdominal aorta (**Figure 2A, Table 2**). Additional findings included diffuse ectasia of the thoracic aorta and stenoses of the renal, iliac, left carotid, and left subclavian arteries. She was treated with prednisone 1 mg/kg/day followed by methotrexate (up to 15 mg/week), achieving clinical and laboratory stability. She has been in remission for at least the past seven years.

**Table 2.** General characteristics of the patients with TAK from the second family.

	**Case 1**	**Case 2**	**Case 3**
Age at diagnosis (years)	33	28	32
Initial manifestations	LL claudication	SAH	LL claudication
Bruits/Murmurs			
Carotid	X	X	X
Aortic	-	-	-
Abdominal	X	-	X
Systolic	X	-	-
Radiological classification (Hata)^30^	?	?	V
Radiological manifestations			
Stenosis			
Left carotid artery	X	X	X
Right carotid artery	-	X	X
Left vertebral artery	-	-	-
Right vertebral artery	-	-	-
Left subclavian artery	X	X	X
Right subclavian artery	-	X	X
Abdominal aorta	X	-	X
Thoracic aorta	-	-	X
Ascending aorta	-	-	-
Right iliac artery	X	-	X
Left iliac artery	X	-	-
Inferior mesenteric artery	-	-	X
Left renal artery	X	-	-
Right renal artery	-	-	-
Occlusions			
Left subclavian artery	-	X	-
Thickenings			
Left carotid artery	-	-	X
Right carotid artery	-	-	-
Left vertebral artery	-	-	-
Right vertebral artery	-	-	-
Brachiocephalic trunk	-	-	X
Left subclavian artery	-	-	X
Right subclavian artery	-	-	-
Abdominal aorta	-	-	X
Descending aorta	-	-	X
Ascending aorta	-	X	-
Dilatations/Aneurysms			
Ascending aorta	-	X	X
Descending aorta	X	-	X
Brachiocephalic trunk	-	-	X

SAH: systemic arterial hypertension; LL: lower limbs.

Her sister, who died at the age of 59, had been diagnosed with TAK at 28 years, during the diagnostic workup for aortic insufficiency. She initially presented with absent upper limb pulses, cardiac murmurs, and elevated inflammatory markers, including erythrocyte sedimentation rate and C-reactive protein. The diagnosis was initially supported by clinical findings and subsequently detailed through imaging, including thoracic CT angiography (**Table 2**). Due to cardiac decompensation, she underwent aortic valve replacement with a bioprosthesis at age 28 and initiated treatment with high-dose prednisone (up to 40 mg/day) and methotrexate (15mg, once weekly), maintained for nine years. The disease proved highly refractory, leading to sequential use of other immunosuppressants: azathioprine (ages 37– 40, ineffective), mycophenolate mofetil (age 40, discontinued after three months due to intolerance), cyclophosphamide (age 44, limited by adverse effects), and long-term leflunomide (20 mg/day, from age 44 for over a decade). Progressive vascular disease with new stenoses required major surgery at age 37, including replacement of the bioprosthetic valve with a mechanical one and placement of a Dacron graft for subclavian occlusions. A subsequent ascending aortic aneurysm necessitated a Cabrol procedure at age 49. Due to persistent inflammatory activity, biologic agents were later introduced— first infliximab (age 52), then tocilizumab (age 54) - but were irregularly administered and ultimately discontinued following recurrent hospitalisations for heart failure. Despite later clinical stability, she died from complications of COVID-19 infection at 59 years of age.

Their brother, currently 58 years old, was diagnosed with TAK, Hata type V, at age 32. His initial presentation included lower limb claudication, carotid and abdominal bruits, and aortic insufficiency. CT angiography revealed extensive vascular involvement, with diffuse thickening of the aorta, brachiocephalic trunk, and left carotid and subclavian arteries, including diffuse parietal calcification and ectasia of the ascending aorta (**Figure 2B**). Multiple stenoses were identified in the descending thoracic and abdominal aorta, carotid, subclavian, right iliac, and inferior mesenteric arteries. Dilation of the thoracic aorta and brachiocephalic trunk was also observed (**Table 2**). Notably, his disease followed an indolent course for over two decades, managed only with supportive medications for blood pressure control (including enalapril, hydrochlorothiazide, and amlodipine) and cardiovascular risk reduction (aspirin and atorvastatin), without requiring corticosteroids or immunosuppressants. However, after a MRA in 2021 revealed new signs of active vascular inflammation, a brief course of methotrexate (10 mg/week) was initiated at age 54 and discontinued at age 57. He is currently in remission and remains under regular outpatient follow-up at our tertiary centre.

### Patients’ perspectives

The patients reported significant challenges related to delayed diagnosis and the chronic course of the disease. The patient from the first family who experienced a stroke emphasised the profound impact of neurological complications on her quality of life but expressed optimism after achieving remission. Her asymptomatic sister initially felt surprised by the diagnosis but expressed gratitude for the proactive screening that enabled early intervention. The surviving siblings from the second family described the emotional burden of witnessing their sister’s severe disease progression and eventual death, which strengthened their commitment to consistent long-term follow-up.

### Discussion of similar published cases

We report five cases of familial TAK from two unrelated families, highlighting the marked heterogeneity in clinical presentation, radiological findings, and prognosis. The phenotypes ranged from oligosymptomatic disease limited to the abdominal aorta to severe, disseminated vasculitis with neurological complications and death. This intrafamilial variability underscores the complex interplay between genetic predisposition and additional modifying factors in the pathogenesis of TAK.

The literature analysis identified 25 cases of familial TAK, which are summarised in **Table 3**.^4–28^

**Table 3.** Described cases of familial TAK.

**Authors**	**Publication (year)**	**Country**	**Family relationship**
Present study	2025	Brazil	Three siblings (2 sisters and 1 brother), 2 sisters
Lavanya et al.^4^	2024	India	Mother and daughter
Hinojosa et al.^5^	2023	Mexico	Monozygotic twins (2 sisters)
Sarikaya et al.^6^	2021	Turkey	2 Sisters
Facchinetti et al.^7^	2018	Italy	Brother and sister
Vargas et al.^8^	2015	Mexico	Brother and sister
Telara et al.^9^	2014	India	Mother and daughter
Deniz et al.^10^	2013	Turkey	Mother and daughter
Morishita et al.^11^	2011	Canada	2 Sisters
Heo et al.^12^	2011	South Korea	2 Sisters
Jeeva et al.^13^	2007	Pakistan	Multiple relatives
Naik et al.^14^	1999	India	2 Sisters
Tsai et al.^15^	1998	Taiwan	2 Sisters
Hong et al.^16^	1992	Korea	Aunt and niece
Tyagi et al.^17^	1991	India	2 Sisters
Khaidarov et al.^18^	1991	Europe	2 Sisters
Valentini et al.^19^	1989	Japan	Mother and daughter
Kodama et al.^20^	1986	Japan	2 Brothers
Isohisa et al.^21^	1984	Japan	2 Sisters
Enomoto et al.^22^	1984	Japan	Monozygotic twins (2 sisters)
Shima et al.^23^	1983	Japan	Aunt and Niece
Makino et al.^24^	1981	Japan	2 Brothers
Numano et al.^25^	1978	Japan	Monozygotic twins (2 sisters)
Zyrianov et al.^26^	1968	Europe	2 Brothers
Hirsh et al.^27^	1964	Japan	2 Sisters
Hermann et al.^28^	1964	Europe	2 Sisters

Consistent with our findings, the literature also demonstrates substantial phenotypic diversity. For example, a report on monozygotic twins described one sibling with typical disseminated disease, while the other showed only limited involvement of the brachiocephalic trunk.^25^ In another report, two brothers presented with nearly identical patterns of renovascular hypertension.^24^ Our cases further expand this spectrum, as the second family exhibited three distinct outcomes: one with a mild and stable course, another with severe progressive disease leading to death, and a third with extensive initial damage who achieved remission following brief medical therapy.

A common theme in both our cases and previous reports is the frequent delay in diagnosis, particularly among non-twin relatives. Many patients are diagnosed at advanced stages, often with established complications such as renovascular hypertension, pulselessness, or claudication.^4,8,10,12,14^ This delay can be attributed to the insidious and nonspecific nature of TAK’s initial symptoms. However, as demonstrated by the asymptomatic sister in our first family, proactive screening can enable early detection. Simple clinical evaluation of first-degree relatives -including four-limb blood pressure measurement and pulse examination -has been advocated and may be crucial for identifying subclinical disease.^12^

The genetic basis of familial TAK remains an area of active investigation. Associations with human leukocyte antigens (HLA), including HLA-B5 and Bw52, have been proposed, but these vary considerably across populations.^5,7,10,23–25^ The occurrence of TAK in consanguineous families has raised the possibility of autosomal recessive inheritance.^8,13^ However, the remarkable variability observed in our families suggests a more complex genetic architecture, potentially involving multiple susceptibility genes with incomplete penetrance, as well as environmental or epigenetic modifiers.

This report adds substantial value to the existing literature by presenting the long-term follow-up of five familial cases of TAK, notably including three affected siblings with markedly divergent clinical outcomes. This observation represents a key contribution, providing rare and concrete evidence of pronounced intrafamilial phenotypic heterogeneity and addressing a significant gap in the literature regarding large and diverse familial clusters. These insights are particularly relevant for clinicians and researchers encountering or investigating familial patterns in this rare large-vessel vasculitis. Nevertheless, our conclusions are limited by the study’s retrospective design and small sample size, precluding definitive statements on heritability. The absence of comprehensive genetic analysis highlights a critical area for future research in these families.

In conclusion, our two-family report demonstrates the profound clinical and prognostic heterogeneity of familial TAK. The diverse outcomes observed emphasise that a familial link does not predict a uniform disease course. These findings underscore the importance of maintaining a high index of suspicion for relatives of patients with TAK, ensuring a prompt evaluation if suggestive symptoms arise**.** Such a strategy may facilitate earlier diagnosis and treatment, potentially mitigating the severe morbidity and mortality associated with this rare vasculitis.

## PRIOR PRESENTATION

A preliminary version of this work was presented as a poster at the 42º Brazilian Congress of Rheumatology, held in Salvador, Brazil, from September 17-20, 2025 (Abstract PT.24.09).
